# Activation dynamics of antigen presenting cells in vivo against *Mycobacterium bovis* BCG in different immunized route

**DOI:** 10.1186/s12865-023-00589-6

**Published:** 2023-11-27

**Authors:** Zhengzhong Xu, Xin Li, Aihong Xia, Zhifang Zhang, Jiaxu Wan, Yan Gao, Chuang Meng, Xiang Chen, Xin-an Jiao

**Affiliations:** 1 Jiangsu Key Laboratory of Zoonosis/Jiangsu Co-Innovation Center for Prevention and Control of Important Animal Infectious Diseases and Zoonoses, Yangzhou University, No. 48 Wenhui East Road, Yangzhou, Jiangsu 225009 China; 2 Key Laboratory of Prevention and Control of Biological Hazard Factors (Animal Origin) for Agrifood Safety and Quality, Ministry of Agriculture and Rural Affairs, Yangzhou University, Yangzhou, 225009 China

**Keywords:** *M. Bovis* BCG, Lymph node, Macrophage, Cytokines, Antigen presentation

## Abstract

**Background:**

Control of Tuberculosis (TB) infection is mainly the result of productive teamwork between T-cell populations and antigen presenting cells (APCs). However, APCs activation at the site of initiating cellular immune response during BCG early infection is not completely understood.

**Methods:**

In this study, we injected C57BL/6 mice in intravenous (i.v) or subcutaneous (s.c) route, then splenic or inguinal lymph node (LN) DCs and MΦs were sorted, and mycobacteria uptake, cytokine production, antigen presentation activity, and cell phenotype were investigated and compared, respectively.

**Results:**

Ag85A-specific T-cell immune response began at 6 days post BCG infection, when BCG was delivered in s.c route, Th17 immune response could be induced in inguinal LN. BCG could induce high level of activation phenotype in inguinal LN MΦs, while the MHC II presentation of mycobacteria-derived peptides by DCs was more efficient than MΦs.

**Conclusions:**

The results showed that BCG immunized route can decide the main tissue of T-cell immune response. Compared with s.c injected route, APCs undergo more rapid cell activation in spleen post BCG i.v infection.

**Supplementary Information:**

The online version contains supplementary material available at 10.1186/s12865-023-00589-6.

## Introduction

Tuberculosis, caused by *Mycobacterium tuberculosis* (*M. tb*) infection, remains a major global public health threat. In 2021, 10.6 million new TB cases have been reported globally, and the number of TB deaths in the HIV-negative population increased to 1.6 million [1]. *Mycobacterium bovis* bacillus Calmette-Guérin (BCG), the only licensed vaccine against TB, is administered intradermally and provides protection against disseminated TB in infants, however, BCG has shown variable efficacy in preventing pulmonary TB in adolescents and adults [2]. Attempts have been made to optimize immunization strategy to enhance T cell immunity and protection efficiency of BCG. A more effective TB vaccine is needed to boost the level of immunity conferred by the current BCG vaccine that provides moderate protection against TB. Both CD4^+^ and CD8^+^ T cell responses are crucial to the control of *M. tb* infection; developing an effective TB vaccine against TB is to induce T cell immunity to immediately control TB infection and eliciting a reservoir of systemic T memory cells [3]. Several strategies have been employed to develop replacement and booster vaccines for BCG against TB; the route of BCG influence systemic and tissue-specific T cell immunity has also been investigated. Administration of BCG by aerosol (a.e) or intravenous (i.v) routes enhanced protection in non-human primates challenged shortly after immunization [4, 5]. High dose i.v administration of BCG induces higher level of antigen-responsive CD4^+^ and CD8^+^ T cell responses in blood, spleen, bronchoalveolar lavage and lung lymph nodes in *Macaca mulatta* model [3]. And another research showed that subcutaneous BCG vaccination can induce tissue-resident memory alveolar macrophages and trained immunity in the lung [6].

T cell responses require antigen presentation cells to process and present *M. tb* Ag to generate peptide-major histocompatibility complex (MHC) complexes. Control of *M. tb* is mainly the result of productive teamwork between T-cell populations and antigen presenting cells (APCs) [7]. Both macrophages (MΦ) and dendritic cells (DCs) are important in the defence against *M. tb* infection, participate in the initiation of early innate immune responses to mycobacterial infections, and APCs process and present TB antigen to both CD4^+^ (via MHC-II) and CD8^+^ T cells (via MHC-I) to trigger specific cellular responses. Once inhaled, *M. tb* particles are readily phagocytosed, processed and presented by alveolar macrophages. Initially, infection of the alveolar macrophages by *M. tb* leads to the activation of multiple functions, including killing intracellular *M. tb* by phagolysosome fusion, respiratory burst, recruitment and activation of additional leukocytes, and regulate immune responses by producing pro- and anti-inflammatory cytokines [8, 9]. Macrophages also play a role in presenting antigens to T cells as well as inducing other antigen-presenting cells to express costimulatory molecules, thereby initiating adaptive immune responses [10]. DCs are specialized in antigen presentation to T cells, and since the major role of MΦs is to ingest and kill invading organisms, they have different roles in the defence against *M. tb*. Previous in vitro studies have investigated the immune responses of APCs against mycobacterial infection, however, there remains a limited understanding for mechanisms by which route of BCG influence APCs activation in vivo.

In this study, to investigate activation dynamics of APCs against BCG infection in different immunized route, we injected BCG via the i.v or s.c route, and compared the immune response of MΦs and DCs in spleen and inguinal LN. Understanding these molecular mechanisms will enhance our ability to prime innate and adaptive immunity against *M. tb* and facilitate the development of improved BCG-based immunization strategies to control TB.

## Methods

### Experimental animals

Six to eight-week-old female C57BL/6 mice were obtained from VITAL RIVER (Beijing, China). The mice were housed at our animal biosafety facilities for strict microbial and parasite control and had free access to standard maintain chow and sterilized water. The animal room was maintained at 22–25 °C and 50% humidity under a 12-h dark-light cycle. The C57BL/6 mice were euthanized by cervical dislocation under isoflurane anaesthesia.

### Cytokine production measurement

C57BL/6 mice were i.v or s.c immunized with 1 × 10^8^ CFU *M. bovis* BCG Pasteur 1173P2 (provided by Dr. Xiaoming Zhang, Institut Pasteur of Shanghai, Chinese Academy of Sciences, Shanghai, China) and sacrificed at 0, 3, 6 and 9 days, and the mice immunized at 0 day were control group. Then the mononuclear cells of spleens and inguinal LNs isolated using Histopaque 1083 (Sigma, USA) were seeded at 1 × 10^6^ cells/well in 96-well plates, and subsequently stimulated with 10 µg/ml Ag85A peptide (amino acids 241–260, SciLight Biotechnology, China), 10 µg/ml Ag85A protein, or 5 µg/ml bovine PPD (Prionics, Switzerland) and incubated at 37 °C in 5% CO_2_. Cellular supernatants were then harvested at 48 h post-stimulation and the concentration of IFN-γ was tested by ELISA kits (BD Biosciences, USA), and Th1/Th2/Th17 cytokines were detected by CBA kits (BD Biosciences, USA).

### T cell subsets analysis

C57BL/6 mice were i.v or s.c immunized as above, the mononuclear cells isolated using Histopaque 1083 were seeded at 1 × 10^6^ cells/well in 96-well plates, and subsequently stimulated with 10 µg/ml Ag85A protein and incubated at 37 °C in 5% CO_2_. The mononuclear cells were stained with APC-anti mouse CD4 or FITC-anti mouse CD8α mAbs, than the ratio of CD4^+^ and CD8^+^ T cells in spleen (i.v) and inguinal LN (s.c) were analysed by FACS.

### In vivo infection assay

C57BL/6 mice were i.v or s.c immunized with 1 × 10^8^ CFUs of rBCG-GFP (provided by Dr. Xiaoming Zhang, Institut Pasteur of Shanghai, Chinese Academy of Sciences, Shanghai, China) and sacrificed at 0, 12, 48 and 96 h, and the mice immunized at 0 h was control group. Then, mice spleen and inguinal LN were removed and perfused with 400 U/ml collagenase type IV (Invitrogen, USA) containing 50 µg/ml DNase I (Invitrogen, USA). Single spleen and LN cell suspensions were prepared, DCs (CD11c ^+^) and MΦs (CD11c^−^ CD11b^+^) were both sorted with an autoMACS separator (Miltenyi Biotec, Germany). Then the percentage of murine LN DCs and MΦs infected with rBCG-GFP was analysed using a FACSCalibur instrument and FlowJo software (FlowJo LLC, USA).

### CFU measurement

C57BL/6 mice were s.c immunized with 1 × 10^8^ CFUs of BCG. LN MΦs were sorted with an autoMACS separator as described above, then pelleted and resuspended in lysis buffer. Ten-fold serial dilutions of these suspensions were then plated on solid Middlebrook 7H10 medium, and colonies were counted after incubation at 37 °C for 2–3 weeks as previously reported [11].

### RT^2^ profiler PCR array

C57BL/6 mice were s.c immunized with 1 × 10^8^ CFUs of BCG in PBS and sacrificed at 0, 12 and 48 h, and the mice immunized at 0 h were control group. Then, inguinal LNs DCs and MΦs were sorted with an autoMACS separator as described above. Cellular RNA was purified with an RNeasy kit (Qiagen, USA) according to the manufacturer’s instructions. Then, cDNA was reverse-transcribed from total RNA using iScript cDNA synthesis kits. The RT^2^ profiler PCR array was carried out by Qiagen according to the manufacturer’s instructions. The P-values were calculated based on Student’s *t*-test of the replicate 2^−ΔΔCt^ values for each gene in the control and treatment groups.

### Antigen presentation assay

C57BL/6 mice were i.v or s.c immunized with 1 × 10^8^ CFU BCG and sacrificed at 2, 4, 12, 24, 48 and 72 h, then splenic and inguinal LNs DCs and MΦs were sorted with an autoMACS separator as described above. For the ex vivo Ag presentation assay, the purified DCs and MΦs were transferred to 96-well microplates and serially diluted in complete medium. DE10 T-cell hybridomas (provided by Dr. Claude Leclerc, Institut Pasteur, Paris, France) were then added at 1 × 10^5^/well and incubation for 24 h, the cellular supernatants were collected and detected for IL-2 content by ELISA kits (BD Biosciences) as previously reported [11].

### Cell phenotype analysis

C57BL/6 mice were s.c immunized with 1 × 10^8^ CFUs of BCG and sacrificed at 0, 12 and 96 h, and the mice immunized at 0 h were control group, then splenic and inguinal LN MΦs were sorted with an autoMACS separator as described above. LN MΦs were then labelled with biotinylated antibodies against CD40, CD80, CD86, and MHC II. Biotin conjugates were visualized using allophycocyanin-conjugated streptavidin, then analysed using a FACSCalibur instrument (BD Biosciences, USA).

### Analysis of transcription level of MHC II and MHC class II transactivator (CIITA)

C57BL/6 mice were s.c immunized with 1 × 10^8^ CFUs of BCG and sacrificed at 0, 24, 48, and 96 h, and the mice immunized at 0 h were control group. LN MΦs (CD11c^−^ CD11b^+^) were sorted using the autoMACS system as described above, then were pelleted and resuspended in lysis buffer. Cellular RNA was purified with RNeasy kit (Qiagen, Valencia, CA, USA) according to the manufacturer’s instructions, and total RNA was reverse-transcribed into cDNA. One-tenth of the resulting cDNA template was used for real-time PCR analysis of transcriptional levels of MHC II, total CIITA, and CIITA type I and type IV. The following primers were shown as Table S1.

### Statistical analysis

All data are expressed as means ± SE. Statistical analysis was performed using Student’s *t*-test. *P* values < 0.05 were considered statistically significant.

## Results

### Kinetics of Ag85A-specific T-cell immune response in BCG infected mice

**Fig. 1:**
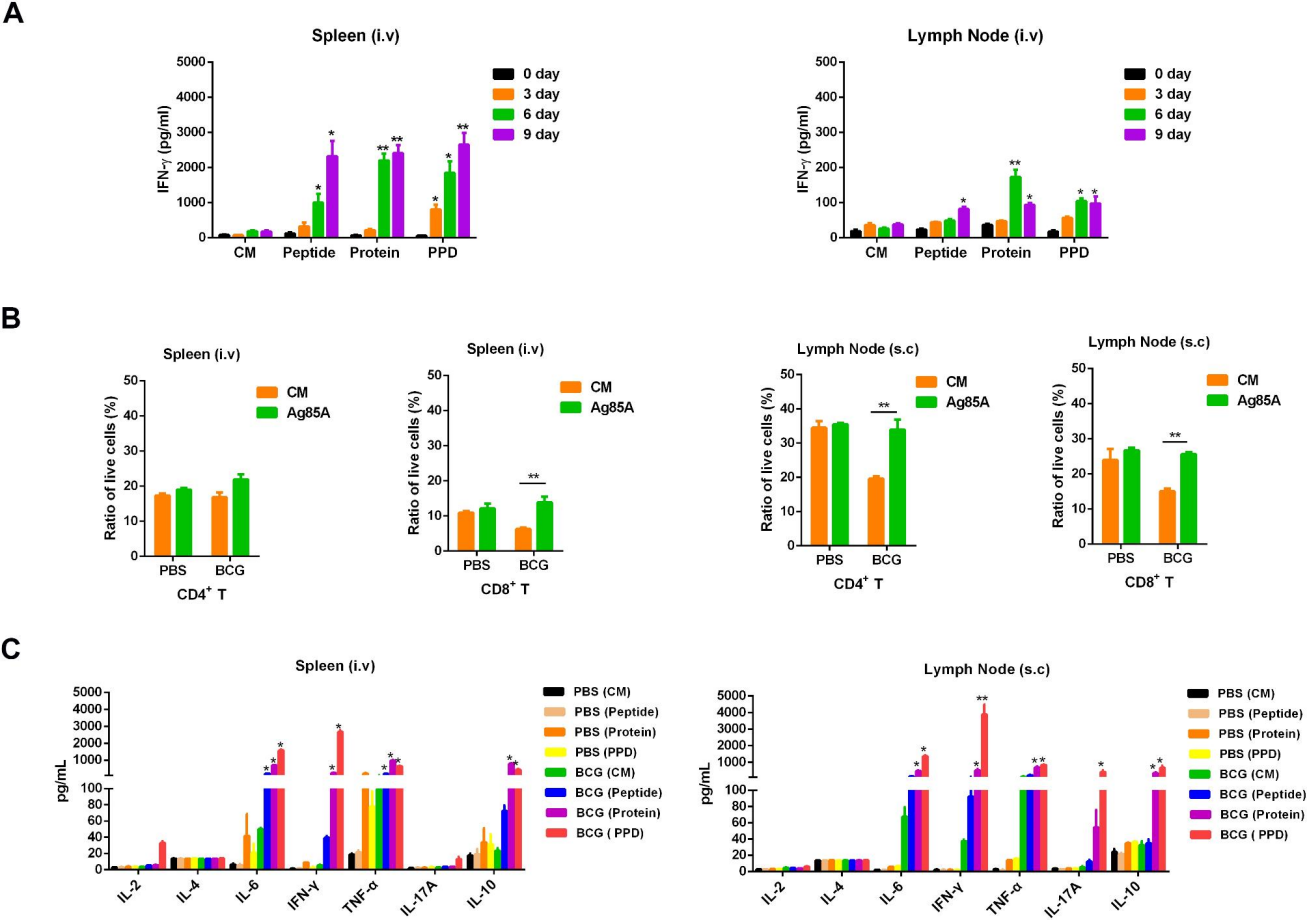
Dynamics of Ag85A-specific T-cell immune response in BCG infected mice. **(A)** Detection of IFN-γ production following BCG infection. C57BL/6 mice (n = 5) i.v infected with 1 × 10^8^ CFU BCG were euthanized by cervical dislocation under isoflurane anaesthesia at 0, 3, 6 and 9 days and the spleens and inguinal LNs were removed. IFN-γ levels in the culture supernatants of splenocytes and inguinal LN cells stimulated with Ag85A peptide, Ag85A protein and Bovine PPD were detected with ELISA. **(B)** Detection of CD4^+^ and CD8^+^ T cell ratio. Ratio of CD4^+^ and CD8^+^ T cells in spleen (i.v) and inguinal LN (s.c) were analysed using FACS stimulated with Ag85A protein. **(C)** Expression profile of Th1 and Th2 type cytokines. Different cytokines in the culture supernatants of splenocytes (i.v) and inguinal LN cells (s.c) stimulated with Ag85A peptide, Ag85A protein and Bovine PPD were detected using FACS. The results are representative of three independent experiments and presented as means ± SEM. Statistical significance was determined using Student’s *t*-test (*P < 0.05, **P < 0.01)

To investigate the kinetics of T-cell immune response against BCG infection, mononuclear cells were separated from mouse spleen and inguinal LN in BCG i.v immunized mice, and stimulated in vitro with Ag85A peptide, Ag85A protein, or bovine purified protein (PPD), respectively, following which the expression level of IFN-γ in culture supernatants were measured. The result showed that Ag85A-specific T lymphocytes in the spleen produced high levels of IFN-γ in Ag85A protein, Ag85A peptide and PPD group, which was a significant increase in Ag85A-specific IFN-γ production by splenic mononuclear cells at 6 and 9 days after BCG injection compared with 0 day control group, however, IFN-γ production was lower in the inguinal LN group (Fig. 1A).

Conversely, we evaluated the kinetics of the Ag85A-specific T-cell immune response in C57BL/6 mice s.c immunized with BCG, in a previous study, and showed that IFN-γ production was 10-fold higher in the inguinal LN [11]. These results suggest that Ag85A-specific T-cell immune response began at 6 days post BCG infection, and the BCG immunization route can decide the main tissue of T-cell immune response. The specific T-cell immune response in inguinal lymph node was higher in BCG s.c immunized mice and spleen in BCG i.v immunization route. As a result, we focus on the immune response of spleen in i.v-injected mice and lymph nodes in s.c injected mice in subsequent research.

Both CD4^+^ and CD8^+^ T cell responses are crucial to the control of *M. tb* infection, the essential role for CD4^+^ and CD8^+^ T cells in immunity to *M. tb* is supported experimentally by the early mortality of mice lacking CD4^+^ and CD8^+^ T cells [12–14]. The CD4^+^ and CD8^+^ T cell proportion of murine spleen and inguinal LN in *M. bovis* BCG immunized mice were analysed via FACS. When BCG was i.v or s.c immunized, the proportion of both CD4^+^ and CD8^+^ T cells in spleen or inguinal LN significantly increased in Ag85A protein stimulated group (Fig. 1B). However, the proportion of both CD4^+^ and CD8^+^ T cells in BCG infected group were lower than PBS control group, possibly due to the rapid recruitment of other mononuclear cells in mouse spleen and inguinal LN.

During *M. tb* infection, Th1 cytokines provide protective function whereas Th2 and Treg cytokines contribute to the pathologic effect, while Th17 cytokines play both protection and pathogenesis [15, 16]. To analyse the expression profile of Th1/Th2 cytokines in murine mononuclear cells against *M. bovis* BCG infection, the mononuclear cells of spleen and inguinal LN were stimulated in vitro with Ag85A peptide, Ag85A protein, or bovine PPD, respectively, then the expression level of a serial of inflammatory secreted cytokines in culture supernatants was measured. When mice were i.v immunized, splenic mononuclear cells showed enhanced production of IL-6, IL-10, IFN-γ and TNF-α in Ag85A peptide and Ag85A protein stimulated groups, however, IL-2 and IL-4 were not detected in splenic mononuclear cells (Fig. 1C). Conversely, when mice were s.c immunized, inguinal LN mononuclear cells showed high level of IL-6, IL-10, IFN-γ and TNF-α, the difference was that IL-17 was highly expressed in inguinal LN mononuclear cells. This suggests that BCG infection induced Th1/Th2 balanced immune response, while when BCG was delivered in s.c route, Th17 immune response could be induced in inguinal LN.

### *M. bovis* BCG infected the inguinal LN antigen presentation cells and induced M1-type activation phenotype

**Fig. 2:**
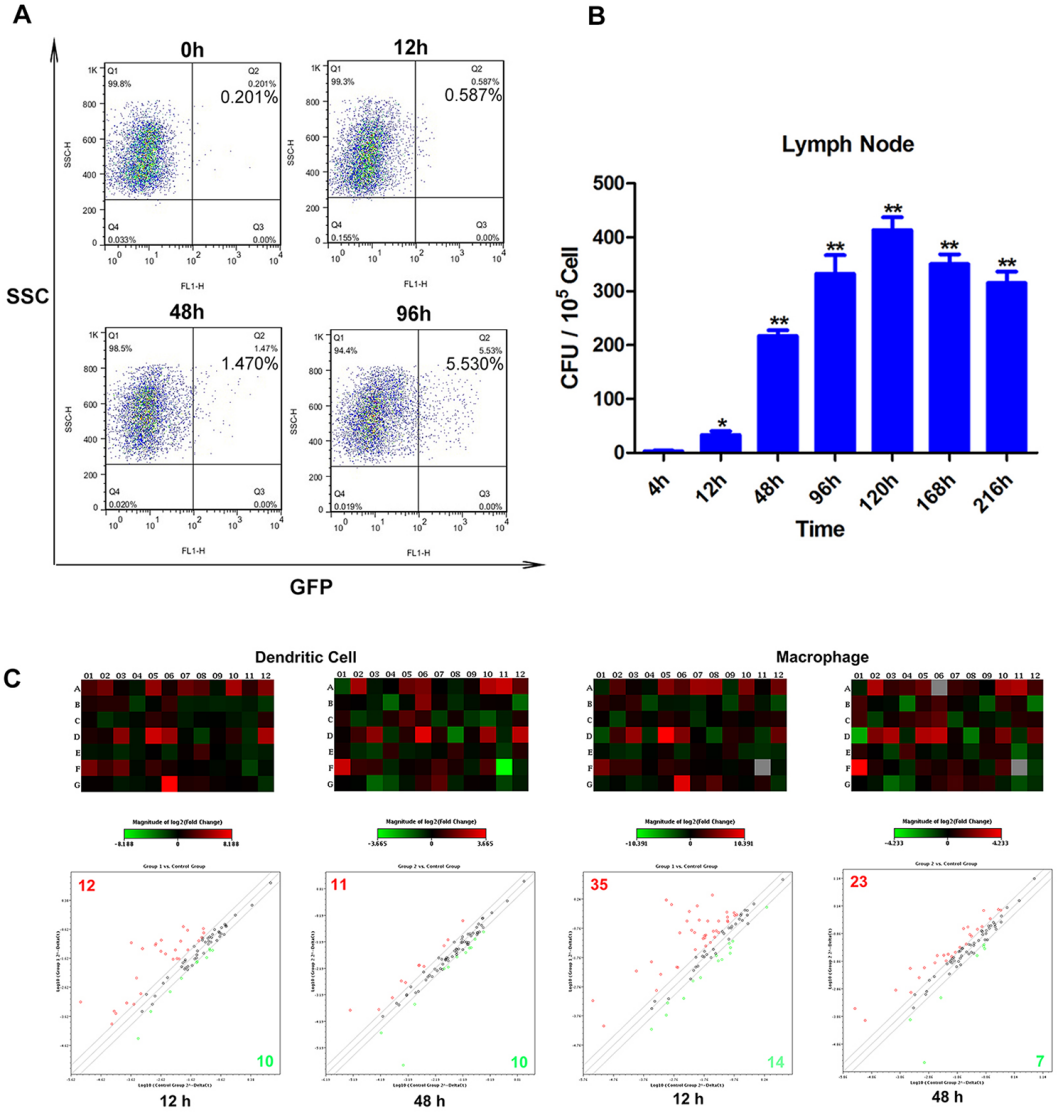
Activation and expression profiles of murine DCs and MΦs against BCG infection. **(A)** Detection of infection rate of LN MΦs against rBCG-GFP. C57BL/6 mice (n = 5) were s.c injected with 1 × 10^8^ CFU rBCG-GFP, LN cells were harvested at different time points, and MΦs were sorted and analysed for the presence of rBCG-GFP. The results are representative of three independent experiments. **(B)** CFU detection of LN MΦs against BCG. Mice (n = 5) were s.c injected with 1 × 10^8^ CFU BCG; BCG in MΦs was quantified in CFUs by culturing on Middlebrook 7H10 agar. **(C)** Transcriptome analysis of murine LN DCs and MΦs against BCG infection. C57BL/6 mice were s.c injected with 1 × 10^8^ CFU BCG cells in PBS. Mice were sacrificed at 12 and 48 h, and inguinal LNs single-cell suspensions were prepared, DCs and MΦs were sorted with an autoMACS separator. The RT^2^ profiler PCR array about antigen presentation pathway associated genes was carried out by QIAGEN, where RNA isolation and quality control were completed. The matrix numbers 1–12 and A-G in heat maps correspond to distinct genes about antigen presentation cells. The results are presented as means ± SEM. Statistical significance was determined using Student’s *t*-test (*P < 0.05, **P < 0.01)

APCs play a dual role in *M. tb* infection, on one hand, they are the main antigen-presenting cells responsible for anti-tuberculosis immunity, while on the other hand, they are a target of *M. tb* and represent a “safe harbour” for the mycobacteria [17]. First, to investigate the ability of DCs (CD11c ^+^) and MΦs (CD11c^−^ CD11b^+^) to engulf *M. bovis* BCG in vivo, we detected the infection rate of rBCG-GFP cells following i.v and s.c infection using FACS. When mice were i.v immunized, both the infection rate of splenic DCs and MΦs reached the peak at 12 h (1.43 and 3.27%, respectively) and decreased at 48 h (1.16 and 1.53%, respectively) (Fig. S1). Conversely, when mice were s.c immunized, the infection rate was different, 1.47% of inguinal LN MΦs exhibited green fluorescence at 48 h, and by 96 h post-injection this proportion increased to 5.53% (Fig. 2A). We also found that 0.4% of LN DCs engulf rBCG-GFP after 12 h of infection, which increased to 2% at 96 h post-injection [11]. Then we determined the number of intracellular *M. bovis* BCG CFUs during the infection. BCG CFUs in inguinal LN MΦs increased until 120 h after s.c administration, followed by a gradually decrease (Fig. 2B), which was similar in LN DCs after s.c administration [11]. These results suggested that both DCs and MΦs can serve as reservoirs of BCG, MΦs have stronger activity of BCG uptake, and BCG remains longer in inguinal LN APCs following s.c infection.

Components of *M. tb* will initiate the antibacterial activity of APCs during infection [18]. Recognition of *M. tb* by APCs induces the production of proinflammatory cytokines, which plays a crucial role in the inflammatory response and the outcome of mycobacterial infections [19, 20]. Finally, to evaluate the transcription levels of cell activation associated genes, we performed RT^2^ profiler PCR array, which contains various 84 genes, including antigen uptake, chemotaxis, cytokines, cytokine receptors and signal transduction-related genes (Table S2). Compared with untreated control group, a 2-fold or greater difference in mRNA expression levels was used as the cut-off to determine the significant regulatory effects on genes involved in inguinal LN DCs and MΦs activation. The results showed that the expression level of up-regulated genes was both higher in inguinal LN DCs and MΦs at 12 h and decreased at 48 h. In inguinal LN DCs, 11 genes were significantly up-regulated (fold-change > 2.0) at both 12 and 48 h after s.c infection with BCG, 13 genes were upregulated only at 12 h, and there were also 17 downregulated genes (fold-change < 0.5) at 12 and/or 48 h. Some chemotaxis, cytokines, cytokine receptors genes, such as CCL2, CCL8, CCL12, CXCL10, FCER1A and FCGR1 were significantly increased in inguinal LN DCs after BCG s.c immunization (Fig. 2C, Table S3). Conversely, in MΦs, 18 genes were significantly up-regulated at both 12 and 48 h after s.c infection with BCG, and 17 and 5 genes were only up-regulated at 12 and 48 h, respectively. And there were also 16 downregulated genes at 12 and/or 48 h (Fig. 2C, Table S4). M1-type inflammatory cytokines TNF-α, IL-6 and IL-12, as well as chemokines CCL2, CCL3, CCL4, CXCL2, CXCL10 and CCL17 were significantly increased in inguinal LN MΦs after early BCG infection. Additionally, antigen presentation-associated genes CD80, CD86 and MHCII, were also significantly up-regulated. Together, these results indicated that BCG could induce M1-type activation phenotype in inguinal LN MΦs, and the activation period of inguinal LN APCs induced by BCG was momentary.

### Activation of antigen presentation activity of DCs and MΦs induced by *M. bovis* BCG in vivo

**Fig. 3:**
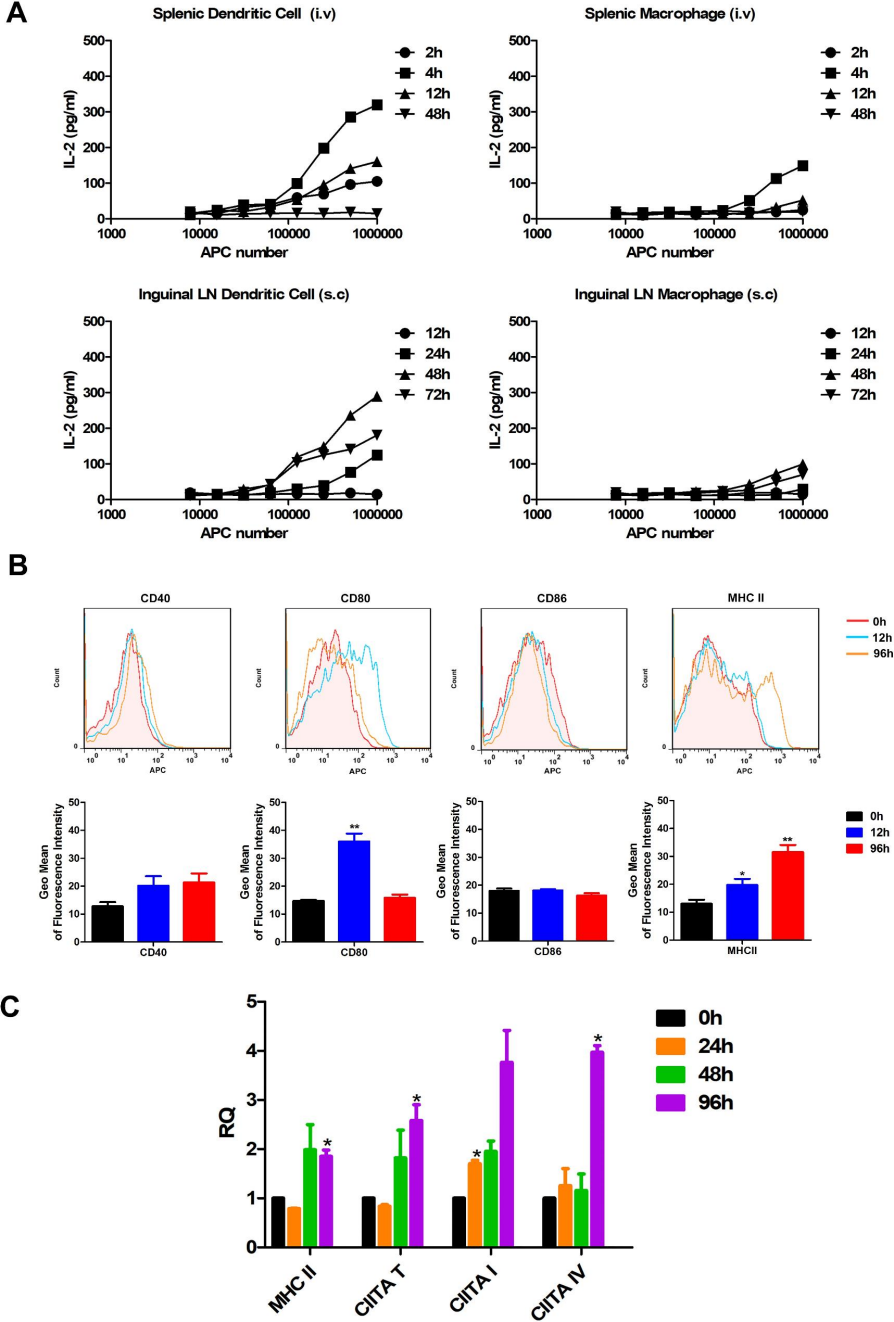
Antigen presentation activity of murine DCs and MΦs against BCG infection. **(A)** Detection of murine DCs and MΦs Ag-presenting activity ex vivo. LN DCs and MΦs from the inguinal LN were harvested and sorted at various time points groups after s.c injection of mice (n = 5) with BCG. Cells were then serially diluted and used to directly stimulate DE10 T-cell hybridomas. In vivo formation of Ag85A peptide-MHC complexes on LN DCs and MΦs from mice injected with Ag85A protein or BCG were estimated by measuring IL-2 production in DE10 T-cell hybridoma culture supernatants ex vivo. **(B)** Analysis of costimulatory molecules on MΦs following BCG infection. Inguinal LNs were obtained at different time points group following s.c infection of mice with 1 × 10^8^ CFU BCG (n = 5), and MΦs were sorted and stained with a panel of mAbs to detect cell-surface expression of CD40, CD80, CD86 and MHC II using FACS. The experiment was repeated at least three times. **(C)** Expression of MHC II and CIITA by MΦs following BCG infection. Inguinal LNs were obtained from mice of four group (n = 5) at different time points following s.c injection of 1 × 10^8^ CFU BCG, transcription levels of MHC II, total CIITA, CIITA types I and IV were analysed using real-time PCR. The results are representative of three independent experiments and presented as means ± SEM. Statistical significance was determined using Student’s *t*-test (*P < 0.05, **P < 0.01)

To investigate the dynamics of Ag85A specific Ag presentation, the splenic and inguinal LN DCs and MΦs were sorted using autoMACS. We tested their capacity to stimulate DE10 T-cell hybridomas at several time points after i.v or s.c injection of mice with Ag85A protein or BCG. When mice were i.v injected with Ag85A protein, high level of IL-2 was detected in the supernatants of splenic DCs after co-cultured with DE10 T-cell hybridomas at 4 h, yet disappeared at 48 h, while splenic MΦs also showed Ag presentation activity at 4 h (Fig. 3A). When mice were s.c injected with Freund’s adjuvant emulsified Ag85A protein, the emerge of IL-2 production of inguinal LN DCs was delayed to 24 h, and peaked at 48 h, low IL-2 level was also detected in the supernatants of inguinal LN MΦs. When mice were s.c immunized with BCG, the highest IL-2 production was only observed in response to DCs from mice infected at 12 h [11], while the Ag presentation activity was not detected in inguinal LN MΦs, and specific Ag presentation activity were also not detected in either splenic DCs or MΦs in BCG i.v immunized mice. Together, these results indicated that Ag-presenting activity was transient on DCs and MΦs in vivo and was initiated earlier in splenic DCs and MΦs with i.v injection route, and suggested that the MHC II presentation of mycobacteria-derived peptides by DCs was more efficient than MΦs.

MHC molecule expression on the cell surface is required for successful antigen presentation [21]; CD80 and CD86 play an important role in enhancing intercellular contacts and increasing T-cell functional activation [22]. The ability of APCs to prime adaptive T cell immunity is primarily dependent on the interaction between the co-stimulatory molecules such as CD40, CD80 and CD86 on APCs and the corresponding ligand on T cells [7]. Inguinal LN DCs undergo functional activation in the early stages of BCG infection [11]. Subsequently, to evaluate the activation of inguinal LN MΦs after BCG s.c infection in vivo, we measured the expression of cell-surface markers involved in T cell interaction in inguinal LN MΦs using FACS analysis. Sorted inguinal LN MΦs were stained with mAbs against cell-surface markers CD40, CD80, CD86 and MHC II. No obvious change in CD40 or CD86 was detected in LN MΦs during infection. However, high levels of CD80 were noted at 12 h after infection, which subsequently decreased by 96 h. MHC II was upregulated in all sorted MΦs following infection from 12 to 96 h (Fig. 3B). Collectively, the up-regulation of selected cell-surface markers showed that LN MΦs were activated during BCG s.c infection in vivo. And we also investigated the expression of cell-surface markers in splenic MΦs, the results showed that the levels of CD40, CD80 and CD86 both increased significantly at 96 h following i.v infection (Fig. S2).

Classical macrophage activation is characterized by high capacity to present antigen, activation of a polarized type I response, and high production of toxic intermediates [23]. CIITA is a master transcriptional regulator of MHC class II molecules. The three distinct promoters pI, pIII and pIV, which drive expression of CIITA types I, III and IV, respectively, regulate CIITA transcription [24]. When mice were s.c infected with BCG, MHC II expression in inguinal LN DCs increased at a relatively slow rate, while the total CIITA transcription rapidly induced at 24 h and CIITA type I declined at 96 h [11]. In this study, the mRNA levels of antigen presentation associated genes were also detected in inguinal LN MΦs against BCG s.c infection. Transcription of MHC II, total CIITA and CIITA type I was significantly induced at 48 h; all these genes significantly increased within 96 h of BCG infection (Fig. 3C). Interestingly, the transcription levels of MHC II and CIITA in splenic DCs and MΦs decreased from 4 h following BCG i.v infection (Fig. S3), although might have been activated at earlier time points.

## Discussion

T cell immunity is essential for containment of *M. tb* infection; most TB vaccines in development elicit effective T cell immune responses [25]. In this study, we found that the Ag85A-specific T-cell immune response in inguinal lymph node was higher in BCG s.c immunized mice and the spleen in BCG i.v immunized mice. This suggests that high dose of i.v BCG would elicit a high frequency of systemic and tissue resident T cells mediating durable protection against *M. tb* infection [3]. We found that when BCG was i.v or s.c administered, the ratio of both CD4^+^ and CD8^+^ T cells was up-regulated in mouse spleen and inguinal LN when stimulated with Ag85A protein.

The Th1/Th2 balance affects the outcomes of TB infection, CD4^+^ T cell cytokine expression pattern was associated with alterations in the systemic levels of Th1 and Th2 cytokines [26]. When mice were i.v or s.c immunized, splenic or inguinal LN mononuclear cells showed enhanced production of IL-6, IL-10, IFN-γ and TNF-α, while IL-17 only expressed highly in inguinal LN mononuclear cells.

Most *M. tb* cells are initially deposited in the liver and spleen, while few reached peripheral LNs [27]. A higher proportion of LN MΦs were infected with rBCG-GFP compared with lymph node DCs [11]. When mice were s.c immunized, 1.47% of inguinal LN MΦs exhibited green fluorescence at 48 h, and by 96 h post-injection increased to 5.53%. Conversely, in i.v immunized mice, both the infection rate of splenic DCs and MΦs peaked at 12 h (1.43 and 3.27%, respectively) and decreased at 48 h (1.16 and 1.53%, respectively). After infection, macrophages killed and reduced the replication of intracellular mycobacteria through multiple strategies [28]. Therefore, we also determined whether murine LN MΦs could control the intracellular mycobacteria growth during infection. CFU measurements showed that BCG increased up to 120 h after s.c administration of BCG to mice, then gradually decreased. The results suggested that BCG can persist infected within the LN MΦs, which serve as the reservoirs of BCG, while LN MΦs could also kill the intracellular mycobacteria.

The early activation of DCs and MΦs against *M. tb* infection has been elucidated [29, 30]. Lymph nodes are crucial organs for the host to initiate T-cell responses, which play an important role in protective immunity against *M. tb* infection [25, 31]. Chemokines are largely responsible for recruitment of inflammatory cells to the site of infection [32]. The number of up-regulated genes in inguinal LN DCs and MΦs were higher at 12 h, and decreased at 48 h. M1-type inflammatory cytokines TNF-α, IL-6, and IL-12 and chemokines CCL2, CCL3, CCL4, CXCL2, CXCL10 and CCL17 were significantly increased in inguinal LN MΦs after early BCG infection, suggesting that BCG can induce M1-type activation phenotype in inguinal LN MΦs, and the activation period of inguinal LN APCs induced by BCG was momentary.

DCs can prime naive T cells and activate them to mature effector T cells via interactions through the upregulated surface molecules [33]. MΦs also play a role in presenting antigens to T cells as well as inducing other antigen-presenting cells to express costimulatory molecules, thereby initiating adaptive immune responses [34]. Jiao et al. have previously investigated the kinetics of Ag-presenting activity ex vivo at various times and detected the formation of MalE peptide/MHC complexes at 2, 4 and 12 h in spleen following rBCG.MalE i.v infection, then disappeared at 48 h. The authors then injected rBCG.MalE via an s.c route and detected MalE peptide/MHC complexes in draining lymph nodes from 1 to 2 days [33]. When mice were i.v or s.c injected with Ag85A protein, high level of IL-2 was detected in the supernatants of splenic DCs and inguinal LN DCs, but the emerge time was delayed in s.c injected route, while the MHC II presentation of mycobacteria-derived peptides by DCs was more efficient than MΦs. However, specific Ag presentation activity was not detected in splenic DCs or MΦs in BCG i.v immunized mice.

Previous studies have indicated that DCs, but not MΦs, undergo functional activation/maturation in the early stages of BCG infection, almost 95% DCs were MHC class II positive in mice receiving BCG, whereas 25% of MΦs were positive in the BCG groups [33]. During infection, no obvious modulation of CD40 and CD86 on LN MΦs was observed. CD80 levels were high at 12 h, but by 96 h, they decreased. All sorted LN MΦs demonstrated up-regulation of MHC II following the initiation of infection from 12 to 96 h. Collectively, the up-regulation of selected cell-surface markers showed that LN MΦs were activated during BCG infection in vivo. In this study, transcription of MHC II, total CIITA, and CIITA type I was significantly induced at 48 h, and these genes significantly increased within 96 h of BCG s.c infection.

In conclusion, this study revealed that the BCG immunization route can decide the main tissue of T-cell immune response. Compared with s.c injected route, DCs and MΦs undergo more rapid mycobacteria uptake, cell activation and antigen presentation in spleen following BCG i.v infection. Our results provided novel insights into the activation profile associated with DCs and MΦs during BCG infection in different routes, which will facilitate the development of improved BCG-based immunization strategies to control TB. *M. tuberculosis* spreads from one person with pulmonary tuberculosis through aerosols, entering the body via the respiratory tract, reaching the lungs where phagocytic cells become infected, and the first line of defense against *M. tuberculosis* is the APCs in lung. It showed that tissue resident memory cells in the lung are implicated in conferring protective immunity against TB with i.v vaccination [3]. Usually, the protective immunity of lung immune cells against TB should be evaluated in aerosol infection route, and we will try to investigate the immune response of lung APCs against TB in further research.

### Electronic supplementary material

Below is the link to the electronic supplementary material.

Supplementary Material 1

## Data Availability

The data and materials that support the findings of this study are available from the corresponding author upon reasonable request.

## Abbreviations

BCG: bacillus Calmette-Guérin
Ag: antigens APCs, antigen presenting cells
*M. tb*: *Mycobacterium tuberculosis*
TB: Tuberculosis
a.e: aerosol
i.v: intravenous
DCs: dendritic cells
MΦ: macrophages
CM: complete medium
PPD: bovine purified protein
s.c: subcutaneously
rBCG: recombinant BCG
CIITA: class II transactivator
MHC: major histocompatibility complex

## References

[1] WHO. : Global Tuberculosis Report 2021. *Available from*: https://wwww.hoint/publications/en/ 2021.

[2] Singh AK, Praharaj M, Lombardo KA, Yoshida T, Matoso A, Baras AS, Zhao L, Srikrishna G, Huang J, Prasad P (2022). Re-engineered BCG overexpressing cyclic di-AMP augments trained immunity and exhibits improved efficacy against Bladder cancer. Nat Commun.

[3] Darrah PA, Zeppa JJ, Maiello P, Hackney JA, Wadsworth MH 2nd, Hughes TK, Pokkali S, Swanson PA 2nd, Grant NL, Rodgers MA, et al. Prevention of Tuberculosis in macaques after intravenous BCG immunization. Nature. 2020;577(7788):95–102. 10.1038/s41586-019-1817-8 PMC7015856 31894150

[4] Barclay WR, Anacker RL, Brehmer W, Leif W, Ribi E (1970). Aerosol-Induced Tuberculosis in Subhuman Primates and the course of the Disease after Intravenous BCG Vaccination. Infect Immun.

[5] Anacker RL, Brehmer W, Barclay WR, Leif WR, Ribi E, Simmons JH, Smith AW (1972). Superiority of intravenously administered BCG and BCG cell walls in protecting rhesus monkeys (Macaca mulatta) against airborne Tuberculosis. Z Immunitatsforsch Exp Klin Immunol.

[6] Jeyanathan M, Vaseghi-Shanjani M, Afkhami S, Grondin JA, Kang A, D’Agostino MR, Yao Y, Jain S, Zganiacz A, Kroezen Z (2022). Parenteral BCG vaccine induces lung-resident memory macrophages and trained immunity via the gut-lung axis. Nat Immunol.

[7] Harding CV, Boom WH (2010). Regulation of antigen presentation by Mycobacterium tuberculosis: a role for toll-like receptors. Nat Rev Microbiol.

[8] Egen JG, Rothfuchs AG, Feng CG, Winter N, Sher A, Germain RN (2008). Macrophage and T cell dynamics during the development and disintegration of mycobacterial granulomas. Immunity.

[9] Shi L, Jiang Q, Bushkin Y, Subbian S, Tyagi S. Biphasic Dynamics of Macrophage Immunometabolism during Mycobacterium tuberculosis Infection. mBio 2019, 10(2). 10.1128/mBio.02550-18 PMC6437057 30914513

[10] Muntjewerff EM, Meesters LD, van den Bogaart G (2020). Antigen Cross-presentation by macrophages. Front Immunol.

[11] Xu Z, Xia A, Li X, Zhu Z, Shen Y, Jin S, Lan T, Xie Y, Wu H, Meng C (2018). Rapid loss of early antigen-presenting activity of lymph node dendritic cells against Ag85A protein following Mycobacterium bovis BCG Infection. BMC Immunol.

[12] Lu YJ, Barreira-Silva P, Boyce S, Powers J, Cavallo K, Behar SM (2021). CD4 T cell help prevents CD8 T cell exhaustion and promotes control of Mycobacterium tuberculosis Infection. Cell Rep.

[13] Patankar YR, Sutiwisesak R, Boyce S, Lai R, Lindestam Arlehamn CS, Sette A, Behar SM (2020). Limited recognition of Mycobacterium tuberculosis-infected macrophages by polyclonal CD4 and CD8 T cells from the lungs of infected mice. Mucosal Immunol.

[14] Yang JD, Mott D, Sutiwisesak R, Lu YJ, Raso F, Stowell B, Babunovic GH, Lee J, Carpenter SM, Way SS (2018). Mycobacterium tuberculosis-specific CD4 + and CD8 + T cells differ in their capacity to recognize infected macrophages. PLoS Pathog.

[15] Lyadova IV, Panteleev AV (2015). Th1 and Th17 cells in Tuberculosis: Protection, Pathology, and biomarkers. Mediators Inflamm.

[16] George PJ, Anuradha R, Kumaran PP, Chandrasekaran V, Nutman TB, Babu S (2013). Modulation of mycobacterial-specific Th1 and Th17 cells in latent Tuberculosis by coincident hookworm Infection. J Immunol.

[17] Pieters J (2008). Mycobacterium tuberculosis and the macrophage: maintaining a balance. Cell Host Microbe.

[18] Sia JK, Rengarajan J. Immunology of Mycobacterium tuberculosis Infections. Microbiol Spectr. 2019;7(4). 10.1128/microbiolspec.gpp3-0022-2018 PMC6636855 31298204

[19] Wang J, Li BX, Ge PP, Li J, Wang Q, Gao GF, Qiu XB, Liu CH (2015). Mycobacterium tuberculosis suppresses innate immunity by coopting the host ubiquitin system. Nat Immunol.

[20] Wang L, Wu J, Li J, Yang H, Tang T, Liang H, Zuo M, Wang J, Liu H, Liu F (2020). Host-mediated ubiquitination of a mycobacterial protein suppresses immunity. Nature.

[21] Wieczorek M, Abualrous ET, Sticht J, Alvaro-Benito M, Stolzenberg S, Noe F, Freund C (2017). Major histocompatibility complex (MHC) class I and MHC class II proteins: conformational plasticity in Antigen Presentation. Front Immunol.

[22] Lim TS, Goh JK, Mortellaro A, Lim CT, Hammerling GJ, Ricciardi-Castagnoli P (2012). CD80 and CD86 differentially regulate mechanical interactions of T-cells with antigen-presenting dendritic cells and B-cells. PLoS ONE.

[23] Marino S, Cilfone NA, Mattila JT, Linderman JJ, Flynn JL, Kirschner DE (2015). Macrophage polarization drives granuloma outcome during Mycobacterium tuberculosis Infection. Infect Immun.

[24] Ghorpade DS, Holla S, Sinha AY, Alagesan SK, Balaji KN (2013). Nitric oxide and KLF4 protein epigenetically modify class II transactivator to repress major histocompatibility complex II expression during Mycobacterium bovis bacillus Calmette-Guerin Infection. J Biol Chem.

[25] North RJ, Jung YJ (2004). Immunity to Tuberculosis. Annu Rev Immunol.

[26] Bewket G, Kiflie A, Tajebe F, Abate E, Schon T, Blomgran R (2022). Helminth species dependent effects on Th1 and Th17 cytokines in active Tuberculosis patients and healthy community controls. PLoS Negl Trop Dis.

[27] Chackerian AA, Alt JM, Perera TV, Dascher CC, Behar SM (2002). Dissemination of Mycobacterium tuberculosis is influenced by host factors and precedes the initiation of T-cell immunity. Infect Immun.

[28] Liu CH, Liu H, Ge B (2017). Innate immunity in Tuberculosis: host defense vs pathogen evasion. Cell Mol Immunol.

[29] Lafuse WP, Rajaram MVS, Wu Q, Moliva JI, Torrelles JB, Turner J, Schlesinger LS (2019). Identification of an increased alveolar macrophage subpopulation in old mice that displays unique inflammatory characteristics and is permissive to Mycobacterium tuberculosis Infection. J Immunol.

[30] Silver RF, Walrath J, Lee H, Jacobson BA, Horton H, Bowman MR, Nocka K, Sypek JP (2009). Human alveolar macrophage gene responses to Mycobacterium tuberculosis strains H37Ra and H37Rv. Am J Respir Cell Mol Biol.

[31] Bogle G, Dunbar PR (2010). T cell responses in lymph nodes. Wiley Interdiscip Rev Syst Biol Med.

[32] Domingo-Gonzalez R, Prince O, Cooper A, Khader SA. Cytokines and chemokines in Mycobacterium tuberculosis Infection. Microbiol Spectr. 2016;4(5). 10.1128/microbiolspec.TBTB2-0018-2016 PMC5205539 27763255

[33] Jiao X, Lo-Man R, Guermonprez P, Fiette L, Deriaud E, Burgaud S, Gicquel B, Winter N, Leclerc C (2002). Dendritic Cells Are Host Cells for Mycobacteria in vivo that trigger Innate and Acquired Immunity. J Immunol.

[34] Hope JC, Thom ML, McCormick PA, Howard CJ (2004). Interaction of antigen presenting cells with mycobacteria. Vet Immunol Immunopathol.

