# Supplementary material for: Activation dynamics of antigen presenting cells in vivo against Mycobacterium bovis BCG in different immunized route
Source: BMC Immunol. 2023 Nov 27;24:48. doi: 10.1186/s12865-023-00589-6 (PMC10683112; doi:10.1186/s12865-023-00589-6)
Supplement: Supplementary file 1 — Supplementary Material 1 [file 12865_2023_589_MOESM1_ESM.docx]

**
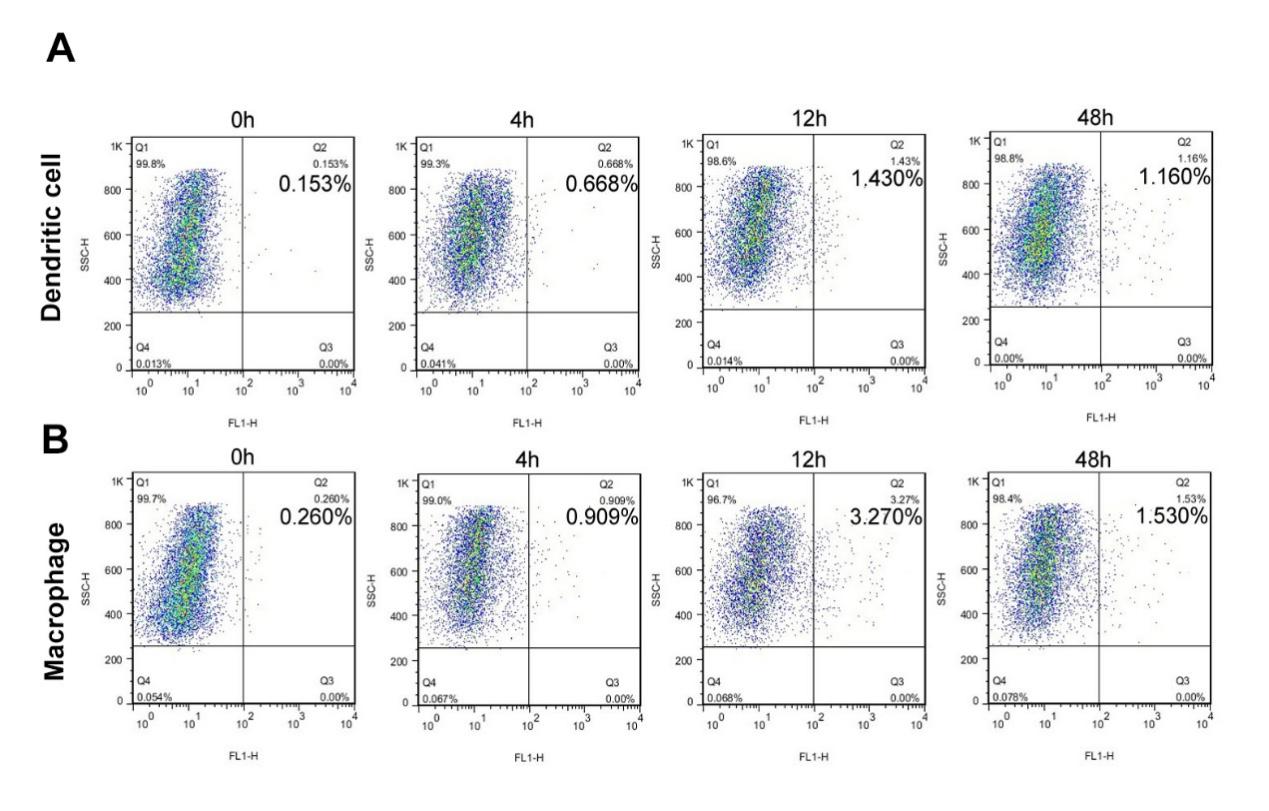
**

**Figure S1. Detection of infection rate of splenic DCs and MΦs against rBCG-GFP.** Groups of mice (n=5) were i.v. injected with 1 × 10^8^ CFU rBCG-GFP, splenic cells were harvested at different time points, and DCs (A) and MΦs (B) were sorted and analyzed for the presence of rBCG-GFP. The results were representative of three independent experiments.


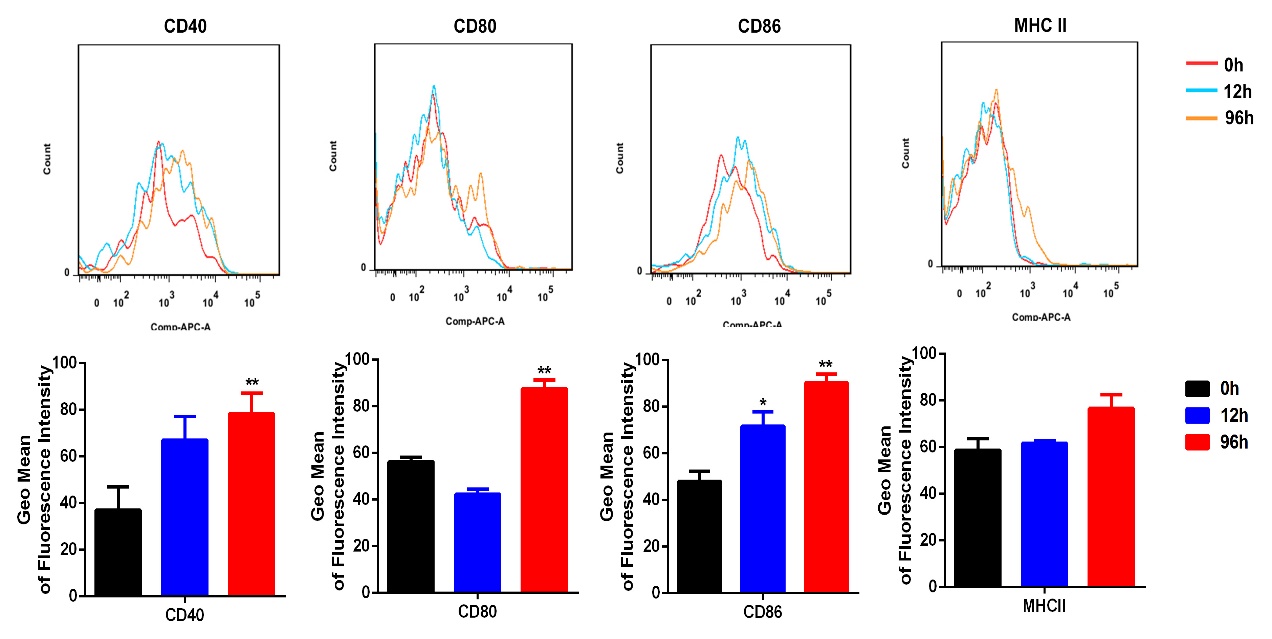


**Figure S2. Analysis of costimulatory molecules on MΦs following BCG infection.** Murine spleens were obtained at different time points group following i.v infection of mice with 1 × 10^8^ CFU BCG (n = 5), and MΦs were sorted and stained with a panel of mAbs to detect cell-surface expression of CD40, CD80, CD86 and MHC II using FACS. The results are representative of three independent experiments and presented as means ± SEM. Statistical significance was determined using Student’s *t*-test (**P* < 0.05, ***P* < 0.01).


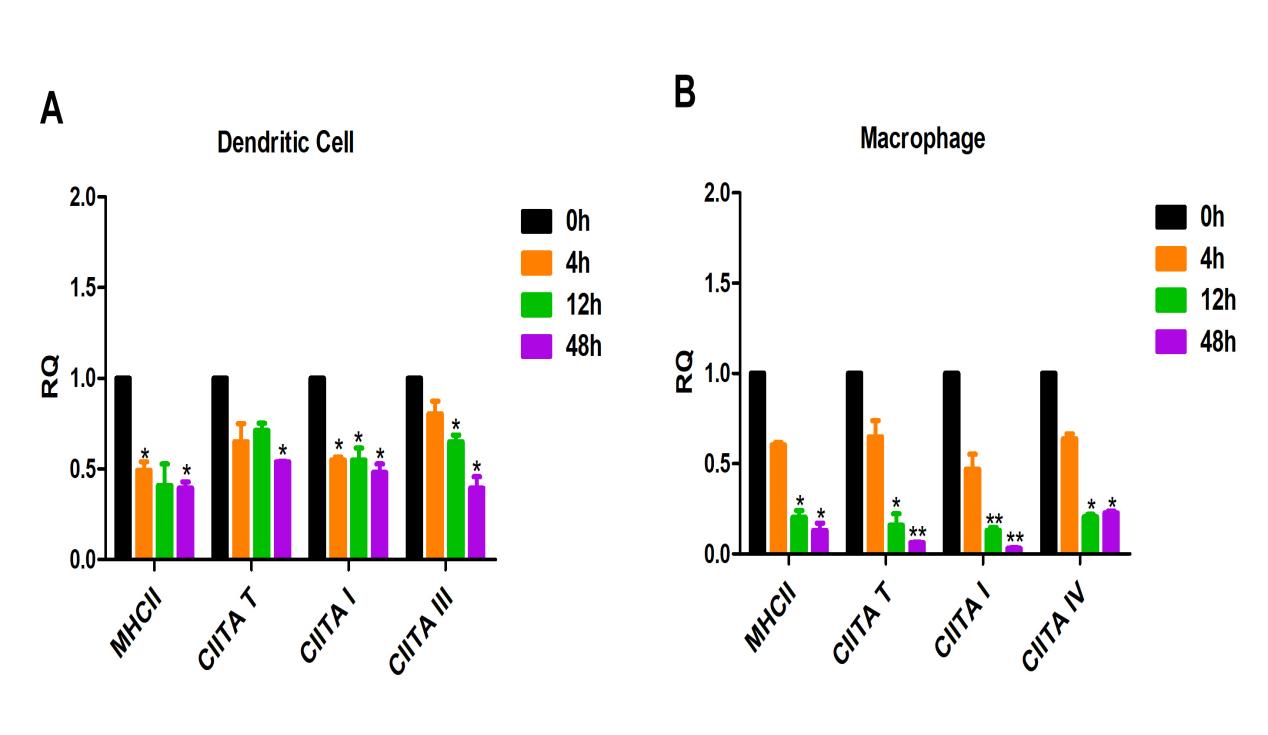


**Figure S3.** E**xpression of MHC II and CIITA by MΦs following BCG infection.** Splenic LNs were obtained from mice of four group (n = 5) at different time points following i.v injection of 1 × 10^8^ CFU BCG, transcription levels of MHC II, total CIITA, CIITA types I and IV were analysed using real-time PCR. The results are representative of three independent experiments and presented as means ± SEM. Statistical significance was determined using Student’s *t*-test (**P* < 0.05, ***P* < 0.01).

**Table S1** Primers for RT-PCR

| **Genes** | **Primers** | |
| --- | --- | --- |
|  |  |  |
| CIITA | F-ACG CTT TCT GGC TGG ATT AGT  R-TCA ACG CCA GTC TGA CGA AGG |  |
| CIITA I | F-AAG AGC TGC TCT CAC GGG AAT  R-GGT CGG CAT CAC TGT TAA GGA |  |
| CIITA III | F-TCT TAC CTG CCG GAG TT  R-GGT CGG CAT CAC TGT TAA GGA |  |
| CIITA IV | F- GAG ACT GCA TGC AGG CAG CA  R-GGT CGG CAT CAC TGT TAA GGA |  |
| MHCII | F-GCG ACG TGG GCG AGT ACC  R-CAT TCC GGA ACC AGC GCA |  |
| GAPDH | F-GCC TTC CGT GTT CCT ACC C  R-TGC CTG CTT CAC CAC CTT C |  |

**Table S2** Detected genes of RT^2^ profiler PCR array

| **Well** | **Gene Symbol** |
| --- | --- |
|  |  |
| A01 | Ccl11 |
| A02 | Ccl12 |
| A03 | Ccl17 |
| A04 | Ccl19 |
| A05 | Ccl2 |
| A06 | Ccl20 |
| A07 | Ccl3 |
| A08 | Ccl4 |
| A09 | Ccl5 |
| A10 | Ccl7 |
| A11 | Ccl8 |
| A12 | Ccr1 |
| B01 | Ccr2 |
| B02 | Ccr3 |
| B03 | Ccr5 |
| B04 | Ccr9 |
| B05 | Cd1d1 |
| B06 | Cd1d2 |
| B07 | Cd2 |
| B08 | Cd209a |
| B09 | Cd28 |
| B10 | Cd33 |
| B11 | Cd36 |
| B12 | Cd4 |
| C01 | Cd40 |
| C02 | Cd40lg |
| C03 | Cd44 |
| C04 | Cd74 |
| C05 | Cd80 |
| C06 | Cd86 |
| C07 | Cd8a |
| C08 | Cdc42 |
| C09 | Cdkn1a |
| C10 | Cebpa |
| C11 | Clec4b2 |
| C12 | Csf1r |
| D01 | Csf2 |
| D02 | Cxcl1 |
| D03 | Cxcl10 |
| D04 | Cxcl12 |
| D05 | Cxcl2 |
| D06 | Cxcr1 |
| D07 | Cxcr4 |
| D08 | Erbb2 |
| D09 | Fas |
| D10 | Fcer1a |
| D11 | Fcer2a |
| D12 | Fcgr1 |
| E01 | Fcgrt |
| E02 | Flt3 |
| E03 | Flt3l |
| E04 | H2-DMa |
| E05 | Icam1 |
| E06 | Icam2 |
| E07 | Ifng |
| E08 | Il10 |
| E09 | Il12a |
| E10 | Il12b |
| E11 | Il16 |
| E12 | Il2 |
| F01 | Il6 |
| F02 | Irf7 |
| F03 | Itgam |
| F04 | Itgb2 |
| F05 | Lrp1 |
| F06 | Lyn |
| F07 | Mif |
| F08 | Nfkb1 |
| F09 | Ptprc |
| F10 | Rac1 |
| F11 | Rag1 |
| F12 | Rela |
| G01 | Relb |
| G02 | Stat3 |
| G03 | Tap2 |
| G04 | Tapbp |
| G05 | Tgfb1 |
| G06 | Thbs1 |
| G07 | Tlr1 |
| G08 | Tlr2 |
| G09 | Tlr7 |
| G10 | Tlr9 |
| G11 | Tnf |
| G12 | Tnfsf11 |
| H01 | Actb |
| H02 | B2m |
| H03 | Gapdh |
| H04 | Gusb |
| H05 | Hsp90ab1 |

**Table S3** Upregulated genes in inguinal LN DCs after BCG infection

|  | **Gene** | **Full Name** | **Fold Change** | | **P-value** | |
| --- | --- | --- | --- | --- | --- | --- |
|  |  |  | **12h** | **48h** | **12h** | **48h** |
| a | CCL12 | Chemokine ligand 12, MCP-5 | 8.52 ± 2.91 | 3.75 ± 0.25 | 0.0232 | 0.0014 |
|  | CCL2 | Chemokine ligand 2, MCP-1 | 33.07 ± 7.61 | 2.77 ± 0.27 | 0.0091 | 0.0038 |
|  | CCL20 | Chemokine ligand 20, MIP-3 | 2.86 ± 0.47 | 2.81 ± 0.35 | 0.0104 | 0.0060 |
|  | CCL7 | Chemokine ligand 7, MCP-3 | 37.52 ± 8.34 | 4.59 ± 0.56 | 0.0085 | 0.0040 |
|  | CCL8 | Chemokine ligand 8, MCP-2 | 2.80 ± 0.20 | 5.57 ± 0.56 | 0.0020 | 0.0025 |
|  | CD1D2 | CD1d2 antigen | 4.13 ± 0.51 | 2.62 ± 2.07 | 0.0043 | 0.0047 |
|  | CXCL10 | Chemokine ligand 10 | 19.90 ± 2.35 | 3.26 ± 0.19 | 0.0026 | 0.0011 |
|  | CXCR1 | Chemokine receptor 1 | 126.14 ± 23.31 | 28.11 ± 4.66 | 0.0057 | 0.0049 |
|  | FCER1A | Fc receptor, IgE | 2.57 ± 0.28 | 3.52 ± 0.24 | 0.0051 | 0.0014 |
|  | FCGR1 | Fc receptor, IgG | 36.90± 8.28 | 4.84 ± 0.41 | 0.0086 | 0.0019 |
|  | IL-6 | Interleukin 6 | 14.35 ± 1.99 | 5.49 ± 0.36 | 0.0037 | 0.0011 |
| b | CCL11 | Chemokine ligand 11 | 4.87 ± 0.42 | 0.31 ± 0.04 | 0.0019 | 0.0005 |
|  | CCL3 | Chemokine ligand 3, MIP-1α | 8.50 ± 1.21 | 0.79 ± 0.08 | 0.0043 | 0.0241 |
|  | CCL4 | Chemokine ligand 4, MIP-1β | 5.66 ± 0.61 | 0.67 ± 0.04 | 0.0028 | 0.0021 |
|  | CCR1 | Chemokine receptor 1, CD191 | 14.35 ± 3.70 | 1.86 ± 0.07 | 0.0123 | 0.0012 |
|  | CCR5 | Chemokine receptor 5, CD195 | 2.35 ± 0.12 | 0.58 ± 0.06 | 0.0013 | 0.0032 |
|  | CD80 | CD80 antigen, B7-1 | 2.51 ± 0.24 | 1.50 ± 0.33 | 0.0043 | 0.0584 |
|  | CSF1R | Colony stimulating factor 1 receptor | 2.39 ± 0.14 | 0.98 ± 0.12 | 0.0018 | 0.3936 |
|  | CXCL2 | Chemokine ligand 2, MIP-2α | 69.90 ± 17.36 | 1.19 ± 0.08 | 0.0103 | 0.0255 |
|  | IL-10 | Interleukin 10 | 3.46 ± 0.68 | 1.27 ± 0.06 | 0.0123 | 0.0082 |
|  | IRF7 | Interferon regulatory factor 7 | 3.63 ± 0.40 | 1.45 ± 0.09 | 0.0038 | 0.0071 |
|  | ITGAM | Integrin alpha M | 7.09 ± 1.27 | 1.19 ± 0.09 | 0.0071 | 0.0328 |
|  | LRP1 | Lipoprotein receptor-related protein 1 | 3.55 ± 0.27 | 0.88 ± 0.10 | 0.0018 | 0.0862 |
|  | TLR2 | Toll-like receptor 2, CD282 | 2.29 ± 0.10 | 0.57 ± 0.05 | 0.0011 | 0.0020 |

a: 11 genes were upregulated both at 12 and 48 h; b: 13 genes were upregulated only at 12 h.

**Table S4** Upregulated genes in inguinal LN MΦs after BCG infection

|  | **Symbol** | **Full Name** | **Fold Change** | | **P-value** | |
| --- | --- | --- | --- | --- | --- | --- |
|  |  |  | **12h** | **48h** | **12h** | **48h** |
| a | CCL12 | Chemokine ligand 12, MCP-5 | 13.50 ± 1.02 | 7.99 ± 1.41 | 0.0011 | 0.0067 |
|  | CCL2 | Chemokine ligand 2, MCP-1 | 56.50 ± 8.36 | 4.49 ± 0.31 | 0.0037 | 0.0013 |
|  | CCL20 | Chemokine ligand 20, MIP-3 | 141.23 ± 21.47 | 69.08 ± 6.01 | 0.0039 | 0.0013 |
|  | CCL3 | Chemokine ligand 3, MIP-1α | 106.11 ± 12.18 | 2.35 ± 0.19 | 0.0022 | 0.0031 |
|  | CCL7 | Chemokine ligand 7, MCP-3 | 37.66 ± 6.04 | 7.32 ± 0.81 | 0.0045 | 0.0027 |
|  | CCR1 | Chemokine receptor 1, CD191 | 24.38 ± 4.20 | 2.50 ± 0.17 | 0.0053 | 0.0021 |
|  | CCR2 | Chemokine receptor 2, CD192 | 3.72 ± 0.34 | 2.56 ± 0.31 | 0.0027 | 0.0064 |
|  | CD80 | CD80 antigen, B7-1 | 3.27 ± 0.51 | 2.21 ± 0.21 | 0.0082 | 0.0051 |
|  | CD86 | CD86 antigen, B7-2 | 2.53 ± 0.26 | 2.91 ± 0.69 | 0.0048 | 0.0204 |
|  | CXCL1 | Chemokine ligand 1 | 6.98 ± 0.52 | 5.49 ± 0.45 | 0.0012 | 0.0017 |
|  | CXCL2 | Chemokine ligand 2 | 14.62 ± 2.26 | 7.70 ± 0.76 | 0.0045 | 0.0021 |
|  | CXCL10 | Chemokine ligand 10 | 64.98 ± 10.37 | 6.87 ± 0.54 | 0.0043 | 0.0014 |
|  | CXCR1 | Chemokine receptor 1 | 55.92 ± 5.02 | 10.77 ± 1.84 | 0.0014 | 0.0058 |
|  | FCER1A | Fc receptor, IgE | 3.47 ± 0.22 | 4.95 ± 0.33 | 0.0013 | 0.0012 |
|  | FCGR1 | Fc receptor, IgG | 23.07 ± 2.98 | 3.39 ± 0.34 | 0.0030 | 0.0033 |
|  | IL-6 | Interleukin 6 | 34.22 ± 6.69 | 21.42 ± 2.63 | 0.0066 | 0.0027 |
|  | RAG1 | Recombination activating gene 1 | 8.38 ± 1.30 | 11.01 ± 1.89 | 0.0051 | 0.0059 |
|  | TLR1 | Toll-like receptor 1, CD281 | 3.26 ± 0.32 | 2.39 ± 0.14 | 0.0034 | 0.0017 |
| b | CCL4 | Chemokine ligand 4, MIP-1β | 81.92 ± 18.60 | 1.58 ± 0.22 | 0.0086 | 0.0234 |
|  | CCR3 | Chemokine receptor 3, CD193 | 2.49 ± 0.24 | 1.20 ± 0.15 | 0.0043 | 0.0747 |
|  | CCR5 | Chemokine receptor 5, CD195 | 4.24 ± 1.12 | 0.93 ± 0.21 | 0.0188 | 0.3163 |
|  | CD33 | CD33 antigen | 4.84 ± 0.64 | 1.60 ± 0.30 | 0.0046 | 0.0372 |
|  | CD44 | CD44 antigen | 2.56 ± 0.30 | 1.52 ± 0.33 | 0.0062 | 0.0562 |
|  | CDKN1A | Cyclin-dependent kinase inhibitor 1A, p21 | 2.39 ± 0.25 | -1.13 ± 0.19 | 0.0052 | 0.0013 |
|  | CLEC4B2 | C-type lectin domain family 4, member b2 | 2.62 ± 0.22 | 1.68 ± 0.27 | 0.0031 | 0.0245 |
|  | CSF1R | Colony stimulating factor 1 receptor, | 2.87 ± 0.28 | 1.65 ± 0.31 | 0.0037 | 0.0347 |
|  | FAS | TNF receptor superfamily member 6 | 3.43 ± 0.41 | 1.38 ± 0.20 | 0.0048 | 0.0418 |
|  | ICAM1 | Intercellular adhesion molecule 1, CD54 | 2.49 ± 0.12 | 1.21 ± 0.15 | 0.0011 | 0.0704 |
|  | IL-12A | Interleukin 12 alpha, IL-12p35 | 3.25 ± 0.70 | 1.48 ± 0.39 | 0.0152 | 0.0854 |
|  | IRF7 | Interferon regulatory factor 7 | 5.05 ± 1.60 | 1.44 ± 0.37 | 0.0241 | 0.0877 |
|  | ITGAM | Integrin alpha M | 5.38 ± 0.37 | 1.36 ± 0.19 | 0.0012 | 0.0399 |
|  | LRP1 | CD91 | 4.21 ± 0.63 | 1.85 ± 0.08 | 0.0062 | 0.0015 |
|  | LYN | Tyrosine-protein kinase | 2.87 ± 0.79 | 1.38 ± 0.13 | 0.0276 | 0.0185 |
|  | TLR2 | Toll-like receptor 2, CD282 | 12.45 ± 2.24 | 1.78 ± 0.21 | 0.0063 | 0.0113 |
|  | TNF | Tumor necrosis factor | 3.88 ± 0.59 | -2.15 ± 0.45 | 0.0068 | 0.0033 |
| c | CCL8 | Chemokine ligand 8, MCP-2 | 1.37 ± 0.17 | 7.34 ± 1.85 | 0.0322 | 0.0136 |
|  | CD1D2 | CD1d2 antigen | -1.43 ± 0.48 | 2.93 ± 0.48 | 0.0064 | 0.0098 |
|  | CD40 | CD40 antigen | 1.61 ± 0.29 | 2.61 ± 0.24 | 0.0324 | 0.0036 |
|  | H2-DMa | Histocompatibility 2, class II | 1.45 ± 0.47 | 2.50 ± 0.46 | 0.1181 | 0.0151 |
|  | RAC1 | RAS-related C3 botulinum substrate 1 | 1.60 ± 0.30 | 2.43 ± 0.21 | 0.0384 | 0.0037 |

a: 18 genes were upregulated both at 12 and 48 h; b: 17 genes were upregulated only at 12 h; c: 5 genes were upregulated only at 48 h.
